# Isoprenaline-Modified Polyethyleneimine as an Efficient Gene Delivery System for Targeted Asthma Therapy and Airway Remodeling Inhibition

**DOI:** 10.34133/bmr.0136

**Published:** 2025-01-31

**Authors:** Jiwon An, Moonhwan Choi, Sol Kim, Hyungkyung Yoon, An-Soo Jang, Sang-Kyung Lee, Taiyoun Rhim

**Affiliations:** ^1^ Department of Bioengineering, College of Engineering, Hanyang University, Seoul 04763, Korea.; ^2^ Department of Internal Medicine, Soonchunhyang University Bucheon Hospital, Bucheon 14584, Korea.; ^3^ Institute of Bioengineering and Biopharmaceutical Research, Hanyang University, Seoul 04763, Korea.

## Abstract

This study introduces a novel gene delivery system, polyethyleneimine modified with isoprenaline (PEI–isoprenaline), to enhance targeted gene delivery in the context of asthma therapy and airway remodeling. In vitro investigations used Beas2B cells to assess the biocompatibility of isoprenaline, PEI–isoprenaline, and small interfering RNA (siRNA)/PEI–isoprenaline complexes, with cytotoxicity evaluations confirming their safety. The transfection efficiency of the siRNA/PEI–isoprenaline complex was scrutinized in THP-1 cells and displayed superior performance in delivering siRNA to cells expressing the β2 adrenergic receptor (ADRB2). In vivo studies used a murine chronic asthma model to evaluate gene delivery to ADRB2-expressing cells in bronchoalveolar fluid and lung tissues. Therapeutic effects were comprehensively assessed through cell analyses, revealing substantial reductions in airway inflammatory cells and fibrosis, particularly in the Arg1 siRNA/PEI–isoprenaline group. The siRNA/PEI–isoprenaline complex exhibited an impressive 80% delivery rate, greatly surpassing the performance of polyethyleneimine 2K (20%). Notably, the complex achieved a substantial 63% reduction in arginase-1 gene expression, validating its therapeutic potential. Noteworthy inhibitory effects on airway hyperresponsiveness were observed, underscoring the complex’s potential as a targeted gene delivery system for asthma treatment. Our findings underscore the promise and effectiveness of the PEI–isoprenaline complex as a gene delivery system, with its demonstrated biocompatibility, transfection efficiency, and therapeutic outcomes, including arginase-1 gene knockdown and mitigation of airway inflammation and fibrosis, indicating it as a promising candidate for advancing asthma therapy and contributing to the understanding and control of airway remodeling in respiratory diseases.

## Introduction

Asthma is a prevalent chronic inflammatory condition of the bronchi that is characterized by an exaggerated bronchoconstrictor response to diverse stimuli, resulting in reversible bronchospasm [1,2].

The underlying pathophysiology involves persistent airway inflammation marked by the infiltration of various cells, including eosinophils, mast cells, and lymphocytes [3]. This inflammation leads to airway hyperresponsiveness that causes the airways to constrict in response to minor stimuli, ultimately resulting in symptoms [4]. Although the airways typically return to their original shape after constriction, repetitive episodes lead to increased thickness, which paves the way for chronic asthma [5]. The remodeling of the smooth muscle layer in response to continuous stimulation manifests as breathing difficulties and asthmatic shock [6].

Airway remodeling is closely tied to asthma severity and the efficacy of asthma medications [7]. Despite the complexity of asthma, current management relies on reliever and controller medications [8]. Controllers are drugs used to manage asthma, primarily by mitigating inflammation in the airways to thwart asthma attacks. Conversely, relievers are medications that swiftly induce bronchodilation to alleviate the symptoms of airway obstruction and promptly halt an ongoing asthma attack [9]. Isoprenaline, the first beta-agonist to be synthesized, is a highly effective bronchodilator and is used in the treatment of slow heart rate and heart block [10].

However, current treatment options can prove insufficient in controlling asthma, especially in individuals with airway remodeling [11], and can culminate in irreversible airway narrowing and pose a life-threatening risk [12].

Arginase-1 (ARG1), a key player in allergic asthma, is overexpressed during abnormal activation of the T helper 2-mediated immune response in asthma [13]. In a normal physiological state, ARG1 hydrolyzes l-arginine to l-ornithine and urea. However, in asthma, as a cytokine of the T helper immune response, ARG1 is highly expressed in epithelial cells, endothelial cells, and macrophages in the lung tissue airways and contributes to airway tissue remodeling [14–17]. ARG1 expression is implicated in increased airway smooth muscle contraction [18], potentially decreasing the arginine availability crucial for nitric oxide (NO) production [19]. The consequent reduction in NO level contributes to heightened airway hyperresponsiveness, a defining feature of asthma [20]. Additionally, ARG1 is associated with increased collagen deposition and fibrosis in the airways [14]. Elevated Arg1 activity could drive fibroblast differentiation into myofibroblasts, exacerbating extracellular matrix component production and fostering airway remodeling [21]. Furthermore, ARG1 activity is linked to the regulation of mucus production in the airways [14,22]. Elevated Arg1 expression contributes to goblet cell hyperplasia, which leads to excessive mucus secretion, a characteristic feature of asthmatic airway remodeling and a contributor to airway obstruction [17,22]. In the context of the immune response, Arg1 plays a role in macrophage polarization, with M2 macrophages expressing high levels of Arg1 associated with tissue repair and remodeling [23]. Imbalances in macrophage polarization contribute to chronic inflammation and remodeling in asthma. Given its pivotal role in various facets of airway remodeling, Arg1 is a potential therapeutic target in asthma [24].

In this investigation, we used isoprenaline as a reliever and ARG1 small interfering RNA (siRNA) as a controller and preventer of airway remodeling in a combination treatment for asthma. To enhance the specificity of gene delivery while preserving the original pharmacological activity of isoprenaline, we synthesized isoprenaline-conjugated polyethylenimine (PEI–isoprenaline) and tested its ability to target cells expressing adrenergic receptor beta with a therapeutic gene while concurrently addressing asthma symptoms by promoting airway dilation—a dual-action therapeutic approach.

In this investigation, we used ARG1 siRNA as a therapeutic gene, targeting Arg1 because of its elevated expression in airway epithelial cells, endothelial cells, and macrophages in lung tissue. Those cells are implicated in mucin secretion and airway tissue remodeling. Essentially, our objective was to examine the feasibility of a novel asthma treatment, assessing whether the inhibition of airway remodeling could reduce asthma symptoms by promoting airway dilation.

## Materials and Methods

### Synthesis of PEI–isoprenaline

PEI–isoprenaline was synthesized in a 2-step conjugation reaction by modifying a previously described method [25,26]. In the first step, ethyl chloroformate (1.26 mmol) and maleimidopropionic acid (1.20 mmol) were dissolved in tetrahydrofuran and triethylamine at 0 °C for 1 h. Isoprenaline (1.42 mmol) was then added, and the mixture was stirred overnight at room temperature. Subsequently, tetrahydrofuran and triethylamine were removed by rotary evaporation under reduced pressure. The remaining residue was dissolved in 100 ml of ethyl acetate, washed with brine, and dried over sodium sulfate. The resulting product was purified by silica gel column chromatography, using acetone/dichloromethane (v/v = 1:9) as the eluting solvent. In the second step, 1 mmol of maleimidopropionic isoprenaline was mixed with 10 μmol of PEI in methanol (pH 6.5 to 7.5) and incubated overnight at room temperature. Subsequently, the reactants underwent dialysis against distilled water for 3 d, using a membrane with a molecular weight cutoff of 1,000 Da to remove any unconjugated isoprenaline derivatives.

### Preparation of the siRNA/PEI–isoprenaline complex

The siRNA/PEI–isoprenaline complex was prepared using the following protocol: Mouse ARG1 siRNA sequences were designed to hinder the translation of ARG1 by binding with ARG1 messenger RNA (mRNA). The sequences were as follows: forward: 5′-CUU UCA GGA CUA GAU AUC AUG GAA G-3′; reverse: 5′-CUU CCA UGA UAU CUA GUC CUG AAA GGA-3′ (Genolution, Seoul, Korea). To track the transfection efficiency, all siRNAs, including the scrambled siRNAs used as negative controls (Integrated DNA Technologies, San Diego, CA, USA), were labeled with cyanine5 (Cy5) using a labeling IT kit (Mirus, Madison, WI, USA) according to the manufacturer’s instructions. Unincorporated Cy5 dye was subsequently removed by gel filtration using G-50 spin columns (Boehringer Mannheim, Indianapolis, IN, USA). The prepared siRNA was then combined with PEI–isoprenaline at various weight ratios (siRNA to PEI–isoprenaline) and incubated at room temperature for 15 min.

### Confirmation of ARG1 siRNA/PEI–isoprenaline complex formation

Formation of the ARG1 siRNA/PEI–isoprenaline complexes was evaluated using agarose gel electrophoresis, dynamic light scattering, and scanning electron microscopy (SEM; NANO SEM 450, FEI, Hillsboro, OR, USA). In brief, ARG1 siRNA and PEI–isoprenaline were combined at various weight ratios (1:0.1, 1:0.5, 1:1, 1:5, 1:10, and 1:20) in nuclease-free water for 20 min at room temperature. Following incubation, the mixtures were electrophoresed on a 1.3% agarose gel containing GelRed (Koma Biotech, Seoul, Korea). A Zetasizer Nano ZS system (Malvern Instruments, UK) was used to analyze the average sizes and zeta potentials of the mixtures. Isoprenaline, PEI–isoprenaline, and ARG1 siRNA/PEI–isoprenaline complexes were applied to carbon tapes at room temperature and allowed to dry overnight. The dehydrated micelles were silver-coated under vacuum, and their shapes and sizes were examined using SEM (NANO SEM 450, FEI, Hillsboro, OR, USA).

### Measurement of β2-adrenergic agonist affinity

The binding affinity of isoprenaline and modified isoprenaline (PEI–isoprenaline) to the β2 adrenergic receptor (ADRB2) was assessed using a PathHunter eXpress ADRB2 G protein-coupled receptor (GPCR) assay kit (DiscoverX, Fremont, CA, USA) according to the manufacturer’s instructions. In brief, 1 × 10^4^ THP-1 cells (ADRB2-expressing cell line) were seeded in a 96-well plate and incubated for 48 h. Subsequently, serially diluted isoprenaline and modified isoprenaline were added to separate samples and incubated for 3 h at 37 °C. After the incubation period, the detection reagent was added, and further incubation was carried out for 1 h at room temperature in the dark. The plate was then read using a microplate reader (Tecan Infinite M200, Tecan Deutschland GmbH, Crailsheim, Germany).

### Heparin competition assay

To assess the stability of the siRNA/PEI–isoprenaline complexes, a heparin competition assay was conducted. In brief, 1 μg of siRNA was mixed with 5 μg of PEI–isoprenaline in 20 μl of a 5% glucose solution and incubated at room temperature for 30 min. Subsequently, various amounts of porcine heparin sodium salt (Sigma-Aldrich, MA, USA) were added, and each mixture was incubated at room temperature for an additional 30 min for the substitution reaction. The final mixtures were analyzed using 1.3% agarose gel electrophoresis.

### MTT cell cytotoxicity assay

The cytotoxicity of the siRNA/PEI–isoprenaline complex in Beas2B cells was determined using the 3-(4,5-dimethylthiazol-2-yl)-2,5-diphenyltetrazolium bromide (MTT) assay (Sigma, St. Louis, MO, USA). All treatments were conducted with 5 × 10^4^ Beas2B cells/well in a 24-well plate. After 72 h of incubation, the MTT reagent was added to each well and incubated for 4 h. The formazan crystals were then solubilized in dimethyl sulfoxide, and the absorbance was measured at 570 nm. Cell viability (%) was calculated using the following equation: cell viability (%) = [(experimental value − blank value)/(control value − blank value)] × 100.

### Transfection of siRNA/PEI–isoprenaline complexes

In vitro THP-1 cells (5 × 10^5^) were seeded in a 24-well plate and incubated for 24 h. For the positive control, Lipofectamine (Life Technologies, Carlsbad, CA, USA) was mixed with siRNA and incubated for 20 min at room temperature. The same amount of siRNA was added and incubated under the same conditions. Three groups were prepared for transfection: a Cy5–siRNA/PEI–isoprenaline complex, naked Cy5–siRNA, and a Cy5–siRNA/Lipofectamine complex. Each sample was added to cell-seeded wells and incubated for 8 h in serum-free culture conditions. Then, the cells were washed with phosphate-buffered saline (PBS), fixed with 4% paraformaldehyde, and counterstained with 4′,6-diamidino-2-phenylindole (DAPI) (Sigma). The fluorescence of the cells was observed using a fluorescence microscope (TE-2000, Nikon Corp., Tokyo, Japan).

### Animal model

Chronic airway inflammation was induced in mice using a 51-d sensitization and airway challenge through a nasal inhalation model. Wild-type BALB/c mice (6 weeks old, Nara Biotech Inc., Seoul, Korea) were intraperitoneally injected with 100 μg of ovalbumin (Sigma) and 20 mg of aluminum hydroxide (Sigma) emulsified in 200 μl of PBS on days 1 and 14 for allergic sensitization. On days 32, 39, and 46, the mice were challenged with 150 μg of ovalbumin in 50 μl of PBS by intranasal injection. The siRNA/PEI–isoprenaline complex and naked siRNA were prepared as 2 μg of siRNA in a total volume of 100 μl of PBS and administered intratracheally on days 32, 33, 34, 39, 40, 41, 46, 47, and 48. Cy5-fluorescence-labeled siRNA was prepared and administered on days 46, 47, and 48. The Institutional Animal Care and Use Committee of Hanyang University approved all of the protocols for animal care and experiments in this study.

### mRNA level analysis

The total lung tissue mRNA levels in each animal group were extracted using the RNAiso RNA isolation reagent (TaKaRa Biomedicals, Seoul, Korea). The extracted RNAs were synthesized into complementary DNA and subjected to reverse transcriptase polymerase chain reaction (RT-PCR). The mouse ARG1 primer sequences were 5′-CTT GCG AGA CGT AGA CCC TG-3′ (forward) and 5′-CGC TTA TGG TTA CCC TCC CG-3′ (reverse). For the housekeeping gene, mouse glyceraldehyde 3-phosphate dehydrogenase, the primer sequences were 5′-ACC ACA GTC CAT GCC ATC AC-3′ (forward) and 5′-TCC ACC ACC CTG TTG CTG TA-3′ (reverse). The amplified PCR products of each group were analyzed through 1.3% agarose gel electrophoresis.

### Bronchoalveolar lavage fluid analysis

Bronchoalveolar lavage fluid (BALF) from each group was collected by tracheal insertion of a 24G endotracheal catheter (Becton Dickinson, Sparks, MD, USA). PBS was administered into the lungs, and the resulting fluid (2 ml) was collected. To concentrate the BALF cells, the collected samples were centrifuged, and each cell pellet was resuspended in 100 μl of PBS. Cell spin centrifugation was performed to attach the cells to glass slides for further experiments. The collected BALF cells underwent immunocytochemistry to confirm the distribution of the injected siRNA. The cells were fixed with 4% paraformaldehyde, washed with Tris-buffered saline, blocked with 5% bovine serum, and then incubated with anti-ADRB2 antibody (Abcam, Cambridge, UK). Fluorescein isothiocyanate (FITC)-labeled anti-rabbit immunoglobulin G (Youngin, Seoul, Korea) was used as a secondary antibody. After DAPI solution (Vector, Burlingame, CA, USA) counterstaining, the BALF cells were observed using a fluorescence microscope. The BALF samples were processed with Diff-Quik staining (Sysmex, IL, USA) to investigate pathological cell changes in each group. The macrophages, lymphocytes, neutrophils, and eosinophils in each group of BALF samples were counted using an optical microscope.

### Animal model lung tissue histology

The lungs of the asthma model mice were harvested after sacrifice. The tissues were fixed in 4% paraformaldehyde, embedded in paraffin, and sectioned into 4-μm slices using a microtome, and then the sections were placed on a glass slide. For immuno-analysis, the slides were deparaffinized, rehydrated, washed with 0.1% NP-40 in Tris-buffered saline, and blocked with Enzo blocking buffer (Enzo, NY, USA). Primary anti-ADRB2 antibody and secondary FITC anti-rabbit antibody were applied to the slides, which then were counterstained with DAPI before being observed under a fluorescence microscope. For collagen level evaluation, Masson’s trichrome staining assay was conducted on lung tissue slides. ImageJ was used for quantitative collagen level analysis. Hematoxylin and eosin staining was also performed to determine the presence of pathological airway conditions in the lung tissues.

### Methacholine test

The methacholine test, conducted by the Department of Internal Medicine at Soonchunhyang University Bucheon Hospital, was used to investigate airway hyperresponsiveness in the animal model. An asthma model was prepared following a previously described protocol. The experimental groups were healthy normal control mice, ovalbumin-challenged asthmatic mice, isoprenaline-treated mice, PEI–isoprenaline-complex-treated mice, and ARG1 siRNA/PEI–isoprenaline-complex-treated mice. The methacholine challenge and measurement of enhanced pause (Penh) values were performed 1 h after drug treatment. Breathing values, calculated from the chamber air pressure, were measured every 10 s for 3 min at methacholine concentrations of 0, 25, 50, and 100 mg/ml. The averages of the calculated Penh values in each group and concentration were the final values for analysis.

### Statistics

The data are presented as mean ± standard deviation of triplicate experiments. The statistical analyses were conducted using SPSS version 27 (SPSS Inc., Chicago, IL, USA). Group comparisons were performed using the Kruskal–Wallis test. When significant differences were observed, the Mann–Whitney *U* test and Tukey’s post hoc test were applied for further pairwise comparisons. A *P* value less than 0.05 was considered statistically significant.

## Results

### Identification and characterization of the PEI–isoprenaline complex

**Fig. 1.**
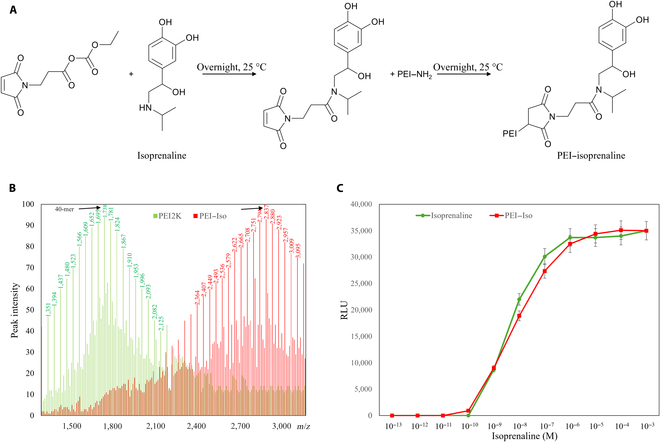
Synthesis and characterization of a conjugate of isoprenaline and polyethylenimine (PEI). (A) Scheme illustrating the synthesis of PEI–isoprenaline using Elfinger et al.’s method [26]. (B) Matrix-assisted laser desorption/ionization time-of-flight (MALDI-TOF) spectra comparing polyethyleneimine 2K (PEI2K) (green) and PEI–isoprenaline (red). Arrows indicate the molecular weight with the highest intensity. (C) Comparison of affinity for the β2 adrenergic receptor between isoprenaline (green) and PEI–isoprenaline (red) in THP-1 cells. PEI–Iso, PEI–isoprenaline; RLU, relative light units.

PEI–isoprenaline was synthesized following Elfinger et al.’s method [26]. The synthetic process and chemical structure are outlined in Fig. 1A. At each step, all reactions underwent confirmation through matrix-assisted laser desorption/ionization time-of-flight mass spectrometry, which also was used for comparisons between polyethyleneimine 2K (PEI2K) and the PEI–isoprenaline complexes. To visualize the distinction between the mass spectra of PEI2K and the PEI–isoprenaline complexes, they are illustrated in a single graph (Fig. 1B). The green line represents the mass spectrometry data of PEI2K, and the red line represents the data of PEI–isoprenaline. Rivera-Tirado and Wesdemiotis [27] reported that the molecular weight of PEI is 43*n* +18. Therefore, the molecular weight of the highest peak of PEI2K indicates that PEI with a molecular weight of 1,738 Da is a polymer consisting of 40 ethylenimine (CH_2_CH_2_NH) units. Furthermore, the peaks are separated by 43 Da, and the overall shape displays a standard normal distribution, suggesting that PEI2K, our study’s starting point, is a mixture of PEIs of different sizes, with 1,738 Da as the representative value.

On the other hand, the molecular weight of PEI–isoprenaline produced by the reaction (Fig. 1B, red line) exhibits a distribution similar to that PEI2K, but its size is shifted by about 1,100 Da. Given that the molecular weight of maleimide-conjugated isoprenaline reacting with PEI–NH_2_ as in Fig. 1A is 371 Da, it can be assumed that 3 molecules of isoprenaline are bound to each molecule of PEI.

To determine whether the chemical attachment of the PEI moiety to isoprenaline affects the potency of isoprenaline, an ADRB2 GPCR assay was conducted. When we analyzed the data, considering that PEI–isoprenaline has 3 isoprenaline moieties per molecule, we found that the chemical attachment of the PEI moiety did not significantly alter the affinity of isoprenaline for ADRB2, as shown in Fig. 1C. The IC_50_ values from this experiment were not significantly different: 35.34 ± 2.42 nM (isoprenaline) and 38.49 ± 2.14 nM (PEI–isoprenaline).

### Characterization of the siRNA/PEI–isoprenaline complex

**Fig. 2.**
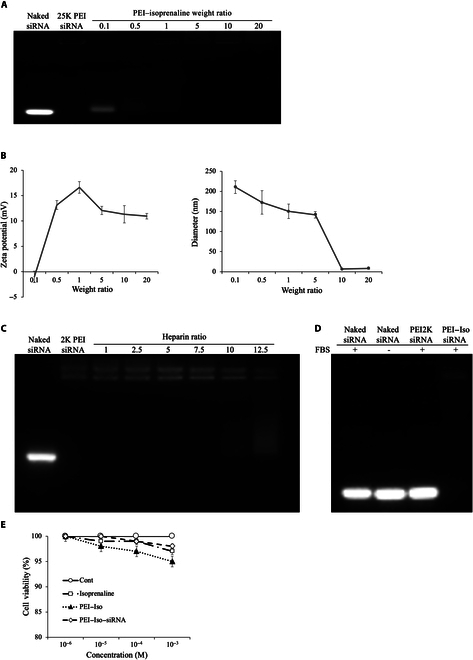
Characterization of the small interfering RNA (siRNA)/PEI–isoprenaline complexes. (A) Identification of the optimal mixing ratio between siRNA and the PEI–isoprenaline carrier via a gel retardation assay. (B) Measurement of zeta potential and mean particle size as a function of the mixing ratio between siRNA and the PEI–isoprenaline carrier using a Zetasizer Nano ZS system. (C) Comparison of competitive binding between the siRNA/PEI–isoprenaline complex and heparin. Above a weight ratio of 1:10, the nucleic acid in the siRNA/PEI–isoprenaline complex began to dissociate. (D) Serum stability of the siRNA/PEI–isoprenaline complex. PEI–isoprenaline is a much more stable complex than PEI2K. (E) The in vitro cytotoxicity of isoprenaline, PEI–isoprenaline, and siRNA PEI–isoprenaline in Beas2B cells was assessed by concentration after 72 h of incubation. The data are presented as mean ± SD, with *n* = 3 replicates for each concentration. FBS, fetal bovine serum; Cont, control.

To verify the formation of the siRNA/PEI–isoprenaline complex, a gel retardation assay was conducted at various weight ratios. As shown in Fig. 2A, naked siRNAs, which have a negative charge, migrated rapidly, whereas the siRNA/PEI–isoprenaline complexes formed stable structures in the well. Comparisons with naked siRNA and PEI2K controls revealed that the most stable and effective condition for siRNA on PEI–isoprenaline was achieved at a weight ratio of 1:5.

The surface charge of the siRNA/PEI–isoprenaline complexes increased significantly with the carrier amount, remaining positive at all tested weight ratios except 1:0.1 (Fig. 2B). Beyond a weight ratio of 1:10, particles could not be formed. The optimal results were observed at a weight ratio of 1:5, with a zeta potential of 12.08 ± 0.81 mV and a particle diameter of 141.68 ± 8.1 nm, revealing a narrow size distribution with a small polydispersity index (0.19 ± 0.03). Complex stability was evaluated in the presence of heparin or serum to assess its suitability for systemic administration.

In the gel retardation assay in heparin conditions, naked siRNA migrated rapidly due to its small size and negative charge, whereas the positive control complex with PEI2K remained stable in the well. At a weight ratio of 1:10, the siRNA/PEI–isoprenaline complex started to disintegrate, and separated siRNAs were detected in the lower region of the gel (Fig. 2C).

Serum compatibility was assessed in reactions with 50% fetal bovine serum for 15 min after 50 min of treatment with 10 μg of heparin. Naked siRNA and the PEI2K complex degraded, but the siRNA/PEI–isoprenaline complex retained its structure, indicating stability superior to that of the PEI2K complex. The results of the heparin competition and serum stability assays confirm the complex stability for systemic administration (Fig. 2D).

### Cell viability and inhibition concentration of the siRNA/PEI–isoprenaline complex

For the in vitro viability assay, the Beas2B normal human lung bronchus epithelial extended cell line was used. The MTT cell cytotoxicity assay compared the cytotoxicity of isoprenaline, PEI–isoprenaline, and the siRNA/PEI–isoprenaline complex. The results revealed no significant difference in cell toxicity among the 3 groups, particularly at the working concentration (less than 10^−6^ M). Thus, all tested groups, including the siRNA/PEI–isoprenaline complex, are well tolerated by Beas2B cells at the concentrations tested in this study. The absence of significant cytotoxicity is a positive outcome, indicating that the treatments do not induce harmful effects on the cells in the given experimental conditions (Fig. 2E).

### In vitro transfection of the siRNA/PEI–isoprenaline complex

**Fig. 3.**
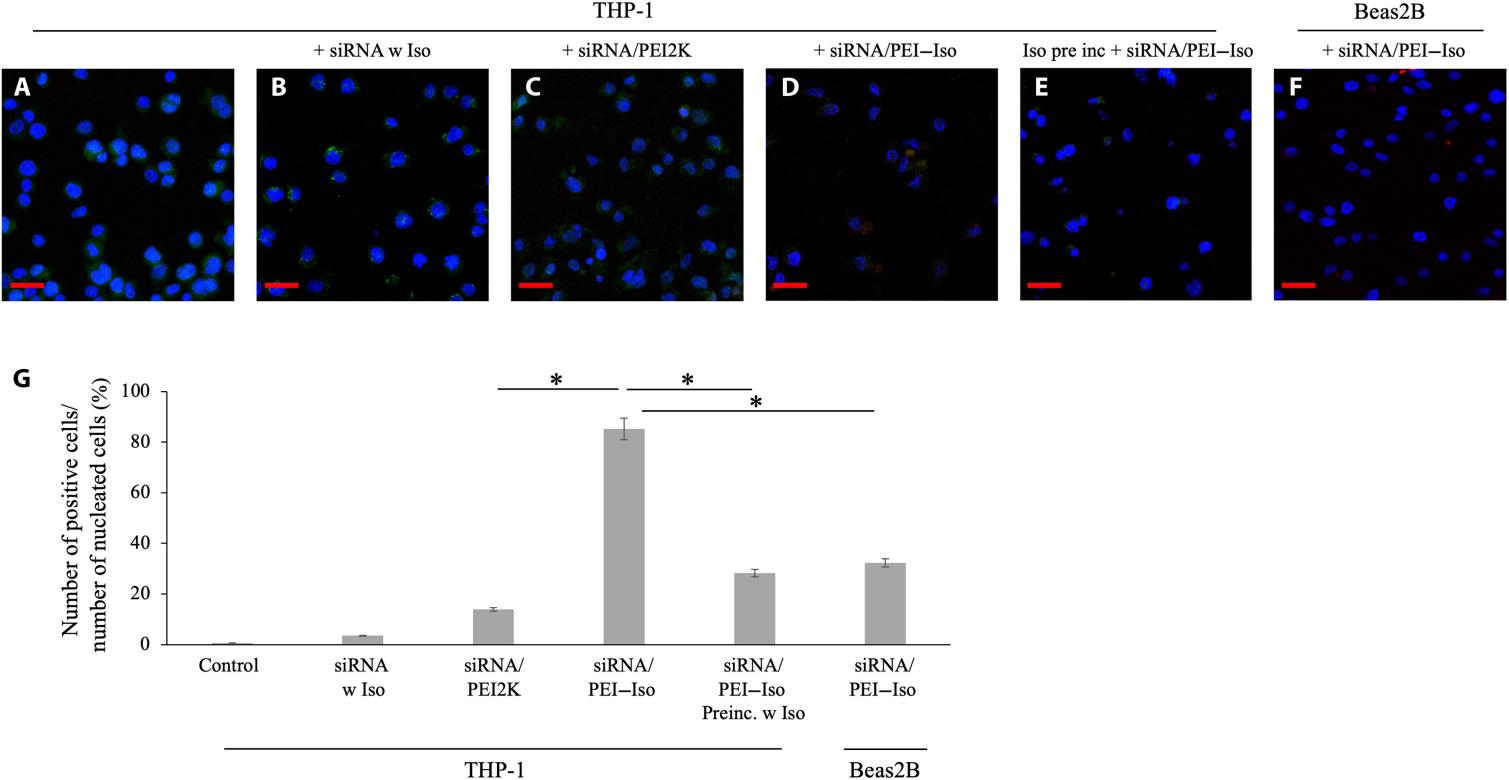
Assessment of gene transfer efficiency in THP-1 cells. The efficiency of gene transfer was assessed in THP-1 cells expressing the β2 adrenergic receptor (ADRB2) and Beas2B cells lacking ADRB2 under standard culture conditions. Cyanine5 (Cy5)-labeled siRNA was transfected into THP-1 cells using isoprenaline (B), PEI2K (C), and PEI–isoprenaline (D). Subsequently, the cells were fixed, and immunofluorescence staining was conducted using rabbit anti-ADRB2 as the primary antibody, followed by incubation with a fluorescein isothiocyanate (FITC)-labeled secondary antibody to visualize ADRB2 expression. THP-1 cells without siRNA served as a positive control for ADRB2 expression (A), and Beas2B cells lacking ADRB2 expression were included as a negative control (F). Pre-treatment with isoprenalineto inactivate the receptor was followed by treatment with siRNA/PEI-isoprenaline, serving as control condition (E). Fluorescent signals in THP-1 and Beas2B cells were quantified using the ImageJ software (each group *n* = 5, *P > 0.05). siRNA w Iso, siRNA with isoprenaline; Preinc. w Iso, preincubated with isoprenaline (E). All experiments were performed independently at least five times and the statistics are shown in (G).

To assess the gene transfer efficiency of our novel carrier, we conducted comprehensive experiments in THP-1 cells, a monocyte cell line expressing ADRB2. Cy5-labeled siRNA was delivered to THP-1 cells in different conditions: siRNA alone, siRNA mixed with isoprenaline, siRNA mixed with PEI2K as the carrier, and siRNA mixed with the newly synthesized PEI–isoprenaline carrier. Post-transfection, the treated cells underwent fixation and immunofluorescence staining using rabbit anti-ADRB2 as the primary antibody and an FITC-labeled secondary antibody to visualize ADRB2 expression. The results, shown in Fig. 3, reveal no discernible fluorescent signal from siRNA in the groups treated with naked siRNA or siRNA mixed with isoprenaline. Using PEI2K as the carrier produced only a minor Cy5 signal in less than 5% of the THP-1 cells. However, the PEI–isoprenaline carrier demonstrated significantly better performance, yielding a robust Cy5 signal in 83% ± 13.7% of THP-1 cells, which indicated efficient siRNA delivery. Additionally, in Beas2B cells lacking ADRB2 expression, the siRNA delivery rate of the PEI–isoprenaline carrier was 17% ± 12.3%, further confirming its efficacy in delivering the therapeutic gene to cells expressing ADRB2. In cells treated with isoprenaline of the same molecular weight 30 min before the experiment, the gene delivery rate was reduced to about one-third (Fig. 3E), confirming that the PEI–isoprenaline gene carrier was delivered by binding to ADRB2. These findings underscore the potential of the PEI–isoprenaline carrier for targeted gene delivery applications.

### In vivo delivery of ARG1 siRNA/PEI–isoprenaline targeting the ADRB2 receptor in animal BALF and lung tissue

**Fig. 4.**
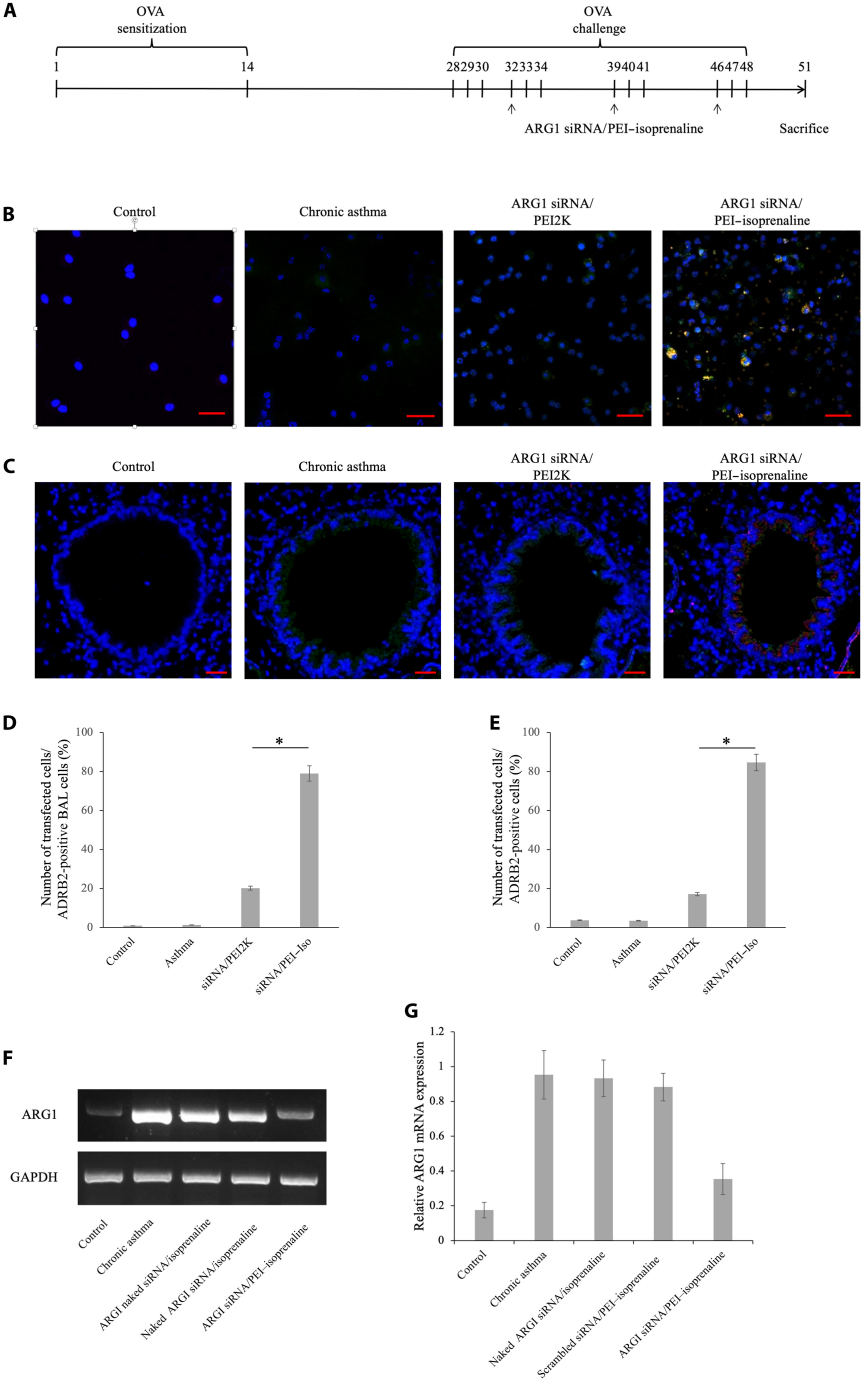
In vivo uptake efficiency of the arginase-1 (ARG1) siRNA/PEI–isoprenaline complex in a mouse model of chronic asthma. (A) Flow diagram illustrating the experimental procedure for the mouse model designed to assess the in vivo uptake efficiency and therapeutic efficacy of the ARG1 siRNA/PEI–isoprenaline complex in the context of chronic asthma. Overlay images of Cy5 (siRNA), FITC (ADRB2), and 4′,6-diamidino-2-phenylindole (DAPI) show the transfection extent of the siRNA/PEI–isoprenaline complex in bronchoalveolar lavage (BAL) cells (B) and airway tissue (C) at ×400 magnification, with a scale bar of 20 μm. The quantification of fluorescence intensity in lung tissue and BAL cells is presented in (D) and (E), with each group consisting of *n* = 6 samples. Statistical significance is denoted by *P < 0.05, and the analysis was performed using the ImageJ software. (F) Semiquantitative reverse transcriptase polymerase chain reaction (RT-PCR) was used to analyze the expression of Arg1 following in vivo delivery of ARG1 siRNA. RT-PCR products were resolved on a 1.3% agarose gel to assess Arg1 expression. (G) Band density analysis of the RT-PCR products (*n* = 3, *P < 0.05). OVA, ovalbumin; GAPDH, glyceraldehyde 3-phosphate dehydrogenase; mRNA, messenger RNA.

The potential of our novel gene delivery system that modifies an airway dilator drug was evaluated in a murine model. The target gene, ARG1, known for its role in NO production and its increased expression in chronic asthma, was subjected to RNA interference. Using the chronic asthma model depicted in Fig. 4A, BALF samples and lung tissues were collected and analyzed after the 51-d experiment.

A specific antibody was used to examine ADRB2 expression in BALF cells and lung tissues from asthmatic and normal mice. The results in Fig. 4B and C reveal little to no ADRB2 expression in the normal lung tissues or bronchoalveolar lavage (BAL) cells. In the chronic asthma model group, a significant increase in ADRB2 expression was observed in most BAL cells, and the lung tissues exhibited elevated expression in bronchi and bronchiole epithelial cells (green).

To assess the efficiency of therapeutic gene delivery, siRNA was labeled with Cy5 for distribution monitoring. siRNAs were delivered to BAL cells and lung tissue using either PEI2K or PEI–isoprenaline as the carrier (Fig. 4B and C). Notably, PEI–isoprenaline demonstrated substantial incorporation into cells expressing ADRB2 (green), unlike PEI2K (Fig. 4D and E). When PEI2K was the carrier, the siRNA delivery rate to ADRB2-expressing cells was approximately 20% in both BAL cells and lung tissues.

However, when PEI–isoprenaline was the carrier, the siRNA delivery rate to ADRB2-expressing cells increased significantly to around 80%, confirming the feasibility of specific gene delivery. These results suggest that our novel carrier, PEI–isoprenaline, holds great promise for efficient and targeted gene delivery in chronic asthma and airway remodeling studies.

### ARG1 knockdown by the ARG1 siRNA/PEI–isoprenaline complex

The efficacy of RNA interference delivery was confirmed through our RT-PCR analysis of lung tissue extracted from the model animals (Fig. 4F and G). In the healthy normal control group, trace amounts of ARG1 RNA were associated with airway remodeling.

Conversely, the asthma-induced group exhibited significantly elevated ARG1 gene expression. The siRNA treatment group comprised 3 subgroups: naked ARG1 siRNA, scrambled siRNA/PEI–isoprenaline, and ARG1 siRNA/PEI–isoprenaline. The naked ARG1 siRNA treatment group did not manifest a substantial decrease in ARG1 gene expression nor did the complex of scrambled siRNA and PEI–isoprenaline, although it showed a tendency toward lower ARG1 RNA expression. However, the ARG1 siRNA/PEI–isoprenaline complex treatment group demonstrated a significant decrease in ARG1 gene expression, with ARG1 expression suppressed by approximately 63% compared with that of the untreated asthma group. These findings indicate that the siRNA/PEI–isoprenaline complex effectively entered the target cells and successfully interfered with the upregulated ARG1 gene, resulting in its suppression.

### Therapeutic effects of the siRNA complex in a chronic asthma model

**Fig. 5.**
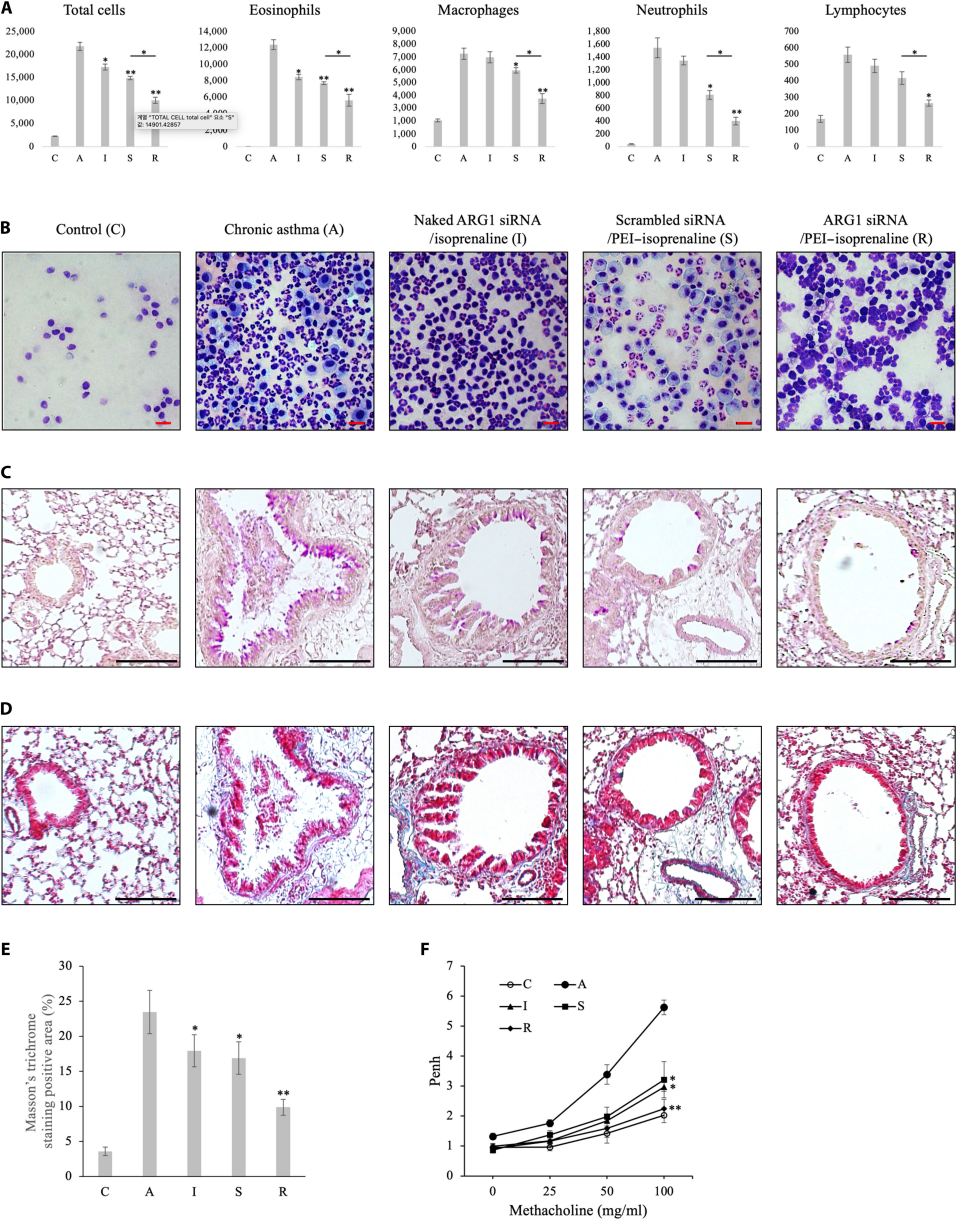
Therapeutic effects of the ARG1 siRNA/PEI–isoprenaline complexes. (A) Bronchoalveolar lavage cell profiles following siRNA treatment. C, healthy normal mouse; A, chronic asthma model; I, naked ARG1 siRNA with isoprenaline; S, scrambled siRNA PEI–isoprenaline complex; R, ARG1 siRNA PEI–isoprenaline complex (*n* = 6; *P < 0.05; **P < 0.005). (B) Photomicrographs of bronchoalveolar lavage cells (magnification ×400, scale bar = 10 μm). (C) Lung tissue sections stained with periodic acid–Schiff (PAS) to detect mucin-producing goblet cells. (D) Masson’s trichrome staining to assess extracellular matrix deposition (scale bar = 50 μm). (E) Quantification of extracellular matrix deposition using the ImageJ software (*n* = 6; *P < 0.05; **P < 0.01). (F) Airway hyperresponsiveness determined by the methacholine test. The ARG1 siRNA PEI–isoprenaline complex group exhibited significantly reduced airway resistance. Moderately reduced airway resistance was observed in the naked ARG1 siRNA with isoprenaline and scrambled siRNA PEI–isoprenaline complex groups (*n* = 6; *P < 0.05; **P < 0.01). Penh, enhanced pause.

Our cell analysis of BALF involved centrifugation and cell immobilization onto slides, followed by hematoxylin and eosin staining and subsequent differential cell counting. This process was replicated in 5 mice per experimental group, and the results are shown in Fig. 5A. The recruitment of airway cells in response to chronic asthma was primarily characterized by an abundance of eosinophils and macrophages. Notably, the administration of Arg1 siRNA/PEI–isoprenaline led to the most substantial reduction in the population of airway inflammatory cells compared with the control groups. Treatment with scrambled siRNA/PEI–isoprenaline was the next most effective in suppressing inflammation, and the combination of isoprenaline and Arg1 siRNA also significantly inhibited the influx of eosinophils into the airways. Both scrambled siRNA/PEI–isoprenaline and Arg1 siRNA/PEI–isoprenaline curtailed the influx of inflammatory cells, including macrophages and eosinophils, into the airways, although the extent of inhibition and alterations in cell morphology differed between them. Specifically, marked changes in macrophage morphology were observed, with the scrambled siRNA/PEI–isoprenaline group exhibiting numerous foamy macrophages akin to those observed in the chronic asthma group. In contrast, the Arg1 siRNA/PEI–isoprenaline group displayed fewer foamy macrophages than the control group.

Similar outcomes were observed in airway tissues, with a substantial influx of inflammatory cells primarily in the bronchioles, accompanied by thickening of the reticular basement membrane region, lamina propria, and submucosa; epithelial detachment; and mucus gland hyperplasia. Arg1 siRNA/PEI–isoprenaline treatment inhibited those symptoms to a degree comparable to the inhibition of inflammatory cell influx. Both the influx of inflammatory cells and bronchiole thickening were inhibited in the order of Arg1 siRNA/PEI–isoprenaline > scrambled siRNA/PEI–isoprenaline > Arg1 siRNA with isoprenaline (Fig. 5C).

Masson’s trichrome staining was used to evaluate the extent of fibrosis, a common feature in chronic asthma. The Arg1 siRNA/PEI–isoprenaline group exhibited the most significant reduction in fibrosis. In contrast, both the scrambled siRNA/PEI–isoprenaline and Arg1 siRNA with isoprenaline groups appeared to suppress fibrosis to a lesser degree, although their effects were similar to each other (Fig. 5E). Furthermore, our investigation involved the assessment of airway hyperresponsiveness induced by methacholine, revealing that isoprenaline treatment exhibited the most pronounced reduction in airway hyperresponsiveness (Fig. 5F, I). Additionally, the combination of isoprenaline and PEI-mediated gene delivery had inhibitory effects on airway hyperresponsiveness (Fig. 5F, S and R). Although there appeared to be a trend indicating that Arg1 siRNA delivery via a gene delivery vehicle that combined isoprenaline with PEI (Fig. 5F, R) resulted in greater inhibition of airway hyperresponsiveness than scrambled siRNA delivered via the same vehicle (Fig. 5F, S), that difference did not attain statistical significance.

## Discussion

Our study presents a comprehensive exploration of a novel PEI–isoprenaline complex as a promising carrier for targeted gene delivery in the context of chronic asthma. Through meticulous identification and characterization, we confirmed the successful synthesis of PEI–isoprenaline, elucidating its chemical structure and molecular weight distribution. The attachment of isoprenaline to PEI occurred at a ratio of approximately 3 molecules of isoprenaline per PEI molecule. The affinity of isoprenaline for ADRB2 GPCR was assessed, and the chemical attachment of the PEI moiety did not significantly alter the potency of isoprenaline. Isoprenaline and PEI–isoprenaline exhibited comparable IC_50_ values, confirming the retention of isoprenaline’s affinity for ADRB2. Furthermore, the formation and characterization of the siRNA/PEI–isoprenaline complex demonstrated its stability and effectiveness, particularly at a weight ratio of 1:5. Its positive surface charge and optimal particle diameter indicated its suitability for systemic administration. Notably, the complex maintained structural integrity in both heparin and serum conditions, highlighting its potential for in vivo applications. In vitro experiments using human lung cells (Beas2B) confirmed the biocompatibility of the siRNA/PEI–isoprenaline complex, with negligible cytotoxicity observed. The in vitro transfection efficiency was assessed in THP-1 cells expressing ADRB2, and the PEI–isoprenaline carrier performed better than the traditional carriers. Moving to in vivo studies in a murine model of chronic asthma, the PEI–isoprenaline complex exhibited remarkable efficiency in delivering ARG1 siRNA to ADRB2-expressing cells in BALF and lung tissues. This specific delivery resulted in a significant knockdown of ARG1 gene expression, indicating successful interference with the gene upregulation associated with airway remodeling in asthma. The therapeutic effects of the siRNA/PEI–isoprenaline complex were evident in the reduction of airway inflammatory cells, the suppression of fibrosis, and the mitigation of airway hyperresponsiveness. Notably, the Arg1 siRNA/PEI–isoprenaline treatment outperformed all control groups, emphasizing the potential clinical relevance of this novel gene delivery system in the context of chronic asthma. Overall, our findings underscore the promising capabilities of the PEI–isoprenaline complex as an effective and targeted gene delivery vehicle, holding significant potential for advancing therapeutic interventions in chronic asthma and related respiratory conditions.

## Data Availability

All data supporting the conclusions are available in the paper. There are no restrictions on data access.
